# Informing, simulating experience, or both: A field experiment on phishing risks

**DOI:** 10.1371/journal.pone.0224216

**Published:** 2019-12-18

**Authors:** Aurélien Baillon, Jeroen de Bruin, Aysil Emirmahmutoglu, Evelien van de Veer, Bram van Dijk

**Affiliations:** 1 Erasmus School of Economics, Erasmus University Rotterdam, Rotterdam, The Netherlands; 2 Ministry of Economic Affairs and Climate Policy, The Hague, The Netherlands; Peking University, CHINA

## Abstract

Cybersecurity cannot be ensured with mere technical solutions. Hackers often use fraudulent emails to simply ask people for their password to breach into organizations. This technique, called phishing, is a major threat for many organizations. A typical prevention measure is to inform employees but is there a better way to reduce phishing risks? Experience and feedback have often been claimed to be effective in helping people make better decisions. In a large field experiment involving more than 10,000 employees of a Dutch ministry, we tested the effect of information provision, simulated experience, and their combination to reduce the risks of falling into a phishing attack. Both approaches substantially reduced the proportion of employees giving away their password. Combining both interventions did not have a larger impact.

## Introduction

Phishing attacks, the attempt to deceptively acquire personal and/or financial information (usernames, passwords etc.) by electronic communication, pose a significant threat for organizations. Social networks are increasingly used in phishing attacks but phishing by emails remains the main risk in an organizational setting. This is due to the relative simplicity of designing and sending phishing emails and its potential to reach many individuals at the same time. The text of a phishing email mostly addresses the recipient with urgency cues, words that invoke feelings of vulnerability or threat, in order to try to force the recipient to act immediately and impulsively. These urgency cues are most deceitful, because they turn attention away from other cues that may potentially help the receiver to recognize a phishing email [1]. Attackers can also trick users into downloading malicious malware, after they click on a link embedded in the email [2]. In recent years, phishing emails have evolved from poorly-designed and untargeted texts into highly personalized and sophisticated messages, which has made recipients more likely to believe that the content is expected and legitimate [3, 4].

In order to cope with increased information security threats and ensure information security, organizations actively take technical security measures [5]. Although these protective mechanisms contribute to improved information security [6], it is rarely enough to entirely rely on them [7]. Organizations that deploy both technical and non-technical protective means are likely to be more successful in protecting against information security risks [8–10]. Organizations create policies and procedures to ensure information security [11]. Although constructing policies and procedures is an essential outset, it is not enough to make sure employees comply with them. Vulnerabilities of the human factor in information security are usually ascribed to non-intentional behavior. Some may simply lack knowledge, skills and abilities to protect themselves against threats, and to comply with existing policies [12].

Is it enough to increase knowledge by providing more information to employees or should companies look for alternative approaches, letting employees experience (a simulated version of) the threat? Cai and Song [13] showed that insurance take-up increased after people played an insurance game. In many cases, people who are victims of phishing fraud never realize that they have been a victim of a phishing attempt or realize it too late, when the extremely negative consequences occur. Letting employees experience (simulated) phishing fraud can complement general information provision. It can make them more receptive to the information.

In a large field experiment, we studied the effect of information provision and experience in reducing the phishing risks. Although existing studies have examined the impact of training and simulation on susceptibility to phishing fraud [14–20], many studies involved role-play activities or lab experiments. In lab experiments, the possibility of phishing email tends to be salient and day-to-day distractions, which increase phishing susceptibility in the real world, are absent. Field experiments avoid these problems but are more difficult to organize. A large-scale field experiment was conducted on students and staff of a university by Mohebzada et al. [21]. The authors found that warnings about phishing risk were not sufficient to prevent users from responding to phishing emails. Other field experiments have been conducted to study the risk factors [22–24] and the effectiveness of phishing exercises in organizations [25] when phishing emails vary in terms of persuasiveness.

Our experiment was conducted at the Dutch Ministry of Economic Affairs, with more than 10,000 subjects who were unaware that they participated in such an experiment. We were thereby able to avoid the many biases which arise in a laboratory experimental setting, and managed to observe actual behavior in a setup that closely mirrors an actual phishing attack. Our experiment consisted of a control and three treatments: information, experience, and both. Information provision aimed at increasing (procedural) knowledge whereas simulated experience could make employees more alert about the threat and more receptive to information. Many authors have argued that combining information and simulated experience leads to stronger effects [14, 15, 19, 20]. Our experimental design allowed us to test for the existence of such synergies. Besides testing the overall effectiveness of our interventions, we were able to study whether gender, age, and employment contract affect an individual’s susceptibility to phishing fraud. This information is of great value to policymakers both within and outside of corporations, as they enable targeted interventions [26].

In the experiment, we sent a phishing email to measure the susceptibility of employees to click on a dubious link and then give away their password. About one third of the subjects clicked on the link in our control treatment and 22% gave their password. Informing subjects about the risks of phishing reduced the proportion of subjects clicking on the link by 7 points and the proportion of password given away by 6 points. A first experience with a phishing email reduced the proportion of subjects clicking on the link by 9 points and of providing their password by 8 points. Combining both the information campaign and the experience intervention did not substantially improve the results with respect to experience alone. Overall, in an organization of the size of the Dutch Ministry of Economic Affairs, letting all employees experience one phishing email can avoid that 800 passwords are given away.

## Conceptual framework

Several models have been proposed to explain phishing susceptibility. They include individual factors (personality, perception, knowledge, motivation) and phishing email characteristics [27, 28]. The latter cannot be influenced by the (victim) organization and we therefore focus on the former.

Due to limited attentional resources, people often rely on automatic or heuristic processing when reading e-mails, increasing the likelihood that people click on links or download malignant software [27]. The level of heuristic processing are influenced by cyber-risk beliefs, which refer to the perception that people have about online threats, and which are influenced by the degree of experience, efficacy, and knowledge that people have on the subject. According to the suspicion cognition automaticity model (SCAM) [27], suspecting a specific email to be a phishing email is directly and indirectly influenced by general cyber-risk beliefs, which include risk perception. Higher risk perception obviously makes people more suspicious about emails but also makes them rely less on heuristic processing and more on systematic processing when reading emails [27]. A deeper, more systematic processing of the information contained in the emails, including the cues signaling phishing threats, makes people better able to detect phishing attacks.

Drawing from the field of education science, a simulated experience is argued to be an effective substitute for learning from an actual experience, especially when simulations provide concrete and emotionally charged experiences [29]. Similar to an experience of an actual phishing email, a simulated phishing email may in this way increase risk perception and subsequently the level of systematic processing and the degree of suspicion applied to future emails. Knowledge, on the other hand, refers to the information the receiver has about the domain of the threat, the ways to detect it and the required actions [26, 28]. Information provision may increase knowledge about the prevalence of phishing attacks and the risks these pose. The information can be about how to detect phishing emails and how to deal with them, which can raise efficacy. Increased knowledge and efficacy together may increase cyber-risk beliefs and subsequently lower the reliance on heuristic processing.

Many papers recommend to incorporate information and experience of phishing emails into one training material [14, 15, 19, 20]. Crucial is to embed the training in the employees’ natural environment [30]. Arachchilage et al. [31] found that combining conceptual knowledge and procedural knowledge is key in enhancing phishing detection and avoidance. A one-time simulated experience is more likely to affect conceptual knowledge (“know that”) than the procedural knowledge (“know how”). Repeated simulated experience could also improve procedural knowledge if sufficient feedback is provided. Information provision can target both. Yet, giving information after employees have received a phishing email can increase the perceived relevance of the information. Information campaigns about procedures to avoid phishing have indeed been found to have greater impact after users have fallen for an attack [32]. From the literature we therefore expect both simulated experience and information provision to be effective, but we also expect their combination to be most effective.

## Method

To test the effectiveness of combining (one-time) experience and information provision on phishing risks, we conducted a large-scale field experiment. The main characteristics of the experiment are outlined below, with further details in S1 Appendix.

### Subjects and design

The subjects of this experiment were 10,929 employees of the Dutch Ministry of Economic Affairs, out of the 12,567 official employees of the Ministry. Reasons for exclusions were mostly technical (e.g., missing information), or related to the rank at the Ministry (Minister, Secretary General…). Details are reported in S1 Appendix. Most subjects were males (60,6%), with an average age of 47 years. Subjects learned that they were part of an experiment only after the experiment was conducted.

We implemented two interventions: information provision (*Info*) and experience (*Exp*). We used a 2x2 design and subjects were divided into four groups of roughly equal size: *Control* (2723 employees), *Info* (2740), *Exp* (2724) and *ExpInfo* (2742).

The experiment was organized around five specific dates *T* ∈ {1, …, 5} (see Table 1 for the exact dates). All four groups received an email that resembled a real phishing email at *T* = 5. In the following, we simply refer to this and all other such emails that we sent a simply phishing email. The first treatment group (*Info*) received an information email in *T* = 2, *T* = 3 and *T* = 4 with information about what phishing is and how it works (*T* = 2), how one can recognize phishing emails (*T* = 3), and what one should do when receiving a phishing email (*T* = 4). The second treatment group (*Exp*) was sent a phishing email at time *T* = 1 and a short debriefing email explaining that the sent email was fake at the end of the same day. No information was given that the emails were part of an experiment. The third treatment group (*ExpInfo*), received both interventions, thus the phishing email and debriefing email at *T* = 1 and the information emails at *T* = 2, *T* = 3 and *T* = 4. One day after the phishing mail at *T* = 5, all four groups received a general debriefing. Table 1 below summarizes the experimental timeline and gives the exact dates of the experiment.

**Table 1:** Experimental timeline.

	*T* = 1	*T* = 2	*T* = 3	*T* = 4	*T* = 5
	05/11/2015	19/11/2015	26/11/2015	03/12/2015	15/12/2015
*Control*					Phishing mail + debriefing
*Info*		Infographic 1	Infographic 2	Infographic 3	Phishing mail + debriefing
*Exp*	Phishing mail + short debriefing				Phishing mail + debriefing
*ExpInfo*	Phishing mail + short debriefing	Infographic 1	Infographic 2	Infographic 3	Phishing mail + debriefing

The choice of the timeline followed several constraints. First, to be in line with our conceptual framework, experience had to precede information. We would then expect subjects from the *ExpInfo* treatment to be more alert about phishing and more receptive to information about it than those from the *Info* treatment, who were not exposed to the first phishing experience. Second, to avoid having one very long email that might discourage readers, the information provided was split between three emails, which were spread over three weeks. Third, we waited one additional week after the end of the *Info* and *ExpInfo* interventions before sending the final phishing email. Sending the final phishing email later was not possible because many employees would be on vacation (Christmas break). Sending it earlier was undesirable. An email on the same day or a day later than the *Info* and *ExpInfo* interventions might be too easily detectable, inflating the measured effectiveness of these treatments. A similar one-week delay was used for instance by Xiong et al. [30] to study the effect of training on phishing detection.

A privacy impact assessment was drawn up to identify potential issues concerning privacy and informed consent. Based on this, the following measures were taken: (1) the analysis was done on anonymized data, the reporting of the results is only on the basis of aggregated data; (2) prior to the experiment the general norm of Information Security System Policy compliance was posted on the intranet; (3) the Employees Council of the Ministry was informed; (4) passwords and other information given by employees were not recorded; and (5) after the experiment, employees were debriefed through an extensive email and were provided with contact details of the researchers. The secretary general and head of internal organization/chief information officer of the ministry gave their (written) approval of the research.

### Group formation

We randomized the subjects at the level of the lowest known organizational unit, henceforth referred to as “basic unit”. In total we had 184 unique basic units, with an average of 61 subjects per basic unit. We have not opted for randomization at the individual level to avoid contamination of the results by intervention spillover effects. With randomization at the individual level, it would have been possible that two subjects working together were divided into different treatment groups. This could have led some subjects to be affected by more than one treatment.

Table 2 shows the distribution of subjects across the four groups. We ran Kruskall-Wallis tests to check if the subjects were equally divided between groups in terms of age, age-groups, gender, employment contract (internal/external), and (the five largest) organizational division (in this paper we refer to them as A, B, C, D, E). Test results showed no statistically significant differences between groups in all variables except, as could be expected, for organizational division (S1 Table). Not all organizational divisions had the same number of basic units (with division A even having only one basic unit) and the size of the basic units varied substantially. We will control for divisional differences in our analysis but it is worth noting that our randomization was successful on all other aspects.

**Table 2:** Descriptive statistics.

Group	N. of subjects	Male	Age						Internal Employee	Organisational division				
			Mean	16-25	26-35	36-45	46-55	>55		A	B	C	D	E
*Control*	2723	60.52%	47.45	2.90%	10.54%	26.07%	35.51%	24.97%	80.21%	–	14.18%	33.79%	19.32%	32.72%
*Info*	2740	61.06%	47.35	2.04%	15.26%	25.69%	28.39%	28.61%	79.34%	9.56%	12.23%	19.60%	13.61%	45.00%
*Exp*	2724	59.99%	47.05	2.86%	13.07%	28.45%	29.22%	26.40%	80.76%	–	10.17%	27.09%	25.33%	37.41%
*ExpInfo*	2742	60.76%	47.31	2.12%	12.65%	26.81%	33.33%	25.09%	80.49%	–	12.47%	26.99%	28.05%	32.49%
**Whole sample**	**10929**	**60.58%**	**47.29**	**2.48%**	**12.88%**	**26.75%**	**31.61%**	**26.27%**	**80.20%**	**2.4%**	**12.26%**	**26.86%**	**21.58%**	**36.91%**

Unfortunately (and out of our control), after the first phishing email at *T* = 1, an online notification was posted for the employees in organizational division C, stating that the phishing email that some employees received was a fake one. Hence the subjects in the *Control* and *Info* groups received this notification as well. This may have affected the results as it created intervention spillover effects within that division. We would then expect treatments effects to be smaller for that division. In what follows, we will always report the analysis with and without this division. Including the division can be expected to give more conservative estimates. Table 3 shows the characteristics of the reduced sample. The distributions of different groups show significant differences on this sample (see S1 Table for Kruskall-Wallis test results).

**Table 3:** Descriptive statistics after the exclusion of division C.

Group	N. of subjects	Male	Age						Internal Employee	Organisational division			
			Mean	16-25	26-35	36-45	46-55	>55		A	B	D	E
*Control*	1803	60.68%	47.45	0.94%	11.15%	29.62%	34.33%	23.96%	70.27%	–	21.41%	29.17%	49.42%
*Info*	2203	56.83%	45.69	2.36%	17.43%	28.92%	28.92%	22.38%	74.58%	11.89%	15.21%	16.93%	55.97%
*Exp*	1986	58.91%	46.14	2.82%	14.20%	31.77%	28.30%	22.91%	74.42%	–	13.95%	34.74%	51.31%
*ExpInfo*	2002	60.24%	46.72	2.35%	13.34%	29.27%	31.72%	23.33%	73.53%	–	17.08%	38.41%	44.51%
**Reduced sample**	**7994**	**59.07%**	**46.46**	**2.15%**	**14.19%**	**29.87%**	**30.69%**	**23.10%**	**73.30%**	**3.28%**	**16.76%**	**29.50%**	**50.46%**

### Procedure

#### Pre-intervention period (all groups)

We first ensured minimum knowledge about the information security policy of the ministry by posting a service notice on the intranet of all five organizational divisions of the ministry prior to the experiment, and visible to all subjects. This message explained the dangers of giving away personal details. Furthermore, the message stated that the Ministry or a division of the Ministry would never ask employees for their password, username etc (S1 Appendix).

#### T = 1: First phishing email: Simulating experience and feedback (groups *Exp* and *ExpInfo*

Subjects from the *Exp* and *ExpInfo* treatments received an imitation of a real phishing email. The subject line was: “Economic Affairs—Mobile Password Recovery System”. This email was sent by the operational management, and subjects were asked to link their account to their mobile phone number in order to recover their password easily if it was lost, or to change it.

The email contained several characteristics enabling receivers to assess the email as being fake/fraudulent, presenting more or less the same level of difficulty as phishing emails that were actually sent at that period. These characteristics were: (1) a misspell in the sender email, (2) inappropriate use of capital letters in the subject line, (3) a change in the logo and logo color, (4) an unusual form of salutation for the Ministry, (5) addressing the receiver in the formal form instead of the informal form, which is normally used, (6) a hyperlink in the email that refers to a vague website with an extension that would normally not be used within the Ministry (.net) and (7) two different but resembling fonts in the main text and the disclaimer (S1 Appendix).

We chose an email subject and sender, which we believed to be equally relevant to most subjects. The link in the email redirected the subjects to a “fake” website (www.mobilepasswordrecoverysystem.net). This website had a very basic design and contained a few elements of the governmental visual design style, with some modifications. In order to link their accounts and phone numbers, subjects were asked to fill in three personal details; (1) username, (2) password, and (3) phone number. After filling in the details, subjects were redirected to a second screen, thanking them for the registration and stating that the registration would be completed within five working days. It was not necessary to fill in all the three personal details. Even if a subject filled in only one field and clicked on “send”, s/he was directed to the second screen that thanked for his/her registration (S1 Appendix).

At the end of the day, all subjects from treatments *Exp* and *ExpInfo* received a short debriefing explaining that the email was an “imitation” email designed to increase awareness for phishing fraud. No information was given that the email was part of an experiment or that there would be follow-up actions (S4 Fig). The debriefing email focused on important it was that all employees contribute to a safer digital environment. By receiving that email, subject could also learn whether they had made a mistake or not. There was no information about how to recognize phishing, nor about how to react. Subjects from the *ExpInfo* treatment would receive such information in the following weeks, as described next.

#### T = 2,3,4: Information provision—Infographics (groups *Info* and *ExpInfo*)

Subjects in treatments *Info* and *ExpInfo* received emails explaining ways to avoid falling for phishing attacks. This information provision occurred in three consecutive weeks, using colorful infographics to maximize the impact of the treatments. The first email explained what phishing is and how it works, the second how receivers could recognize them, and the third what actions receivers should undertake when they receive a phishing email (S1 Appendix). The infographics were designed in a way that (a) makes the subjects understand the risks, (b) keeps the message simple and short, (c) provides clear actionable items that subjects can easily adopt, and (d) uses story-based graphics as suggested by Kumaraguru et al., 2007 and Sheng et al., 2007 [14, 15].

#### T = 5: Second phishing email (all groups)

All subjects of the four groups received a (second) phishing email forty days after the subjects in treatments *Exp* and *ExpInfo* had received a phishing email and twelve days after the subjects from the *Info* and *ExpInfo* treatments had received the last infographics. The second phishing email resembled the first one in terms of looks, length, and recognizable characteristics of phishing mails. Subjects from the *Info* and *ExpInfo*) treatments who would apply the recommendations they received in the infographics should recognize it as a phishing email.

This email was sent by the IT department of the Ministry (with a misspelling in the sender address: helpdesk@dlctu.nl instead of helpdesk@dictu.nl). Subjects were told that they had reached their maximum storage limit of Outlook and the limit had to be raised via a hyperlink to www.verhooogjeopslaglimiet.net, which can be translated as www.increaseyourstoragelimit.net (S1 Appendix). The email asked for an immediate action of the subjects. If they clicked on the link in the email, they were directed to the website. The website they would reach was again basic, with some visuals of Outlook Exchange, subjects were told that by filling in e-mail, username and password, limits could be raised up to 8 GB. If the subject indeed filled in the details, a pop-up screen was shown, stating that the registration was being processed and that it would be completed within five workdays (S6, S7 and S8 Figs).

#### Post-intervention period—Debriefing (all groups)

All subjects received a general debriefing the day after receiving the (second) phishing email. In this elaborate debriefing the subjects were told that the phishing email(s) and information mails were part of an experiment. They were given information about; (1) the cause and purpose of the research, (2) the design of the research, (3) which precautions had been taken in order to respect the privacy of employees and to protect (personal) details, and (4) where subjects could submit other questions and/or remarks.

Furthermore, they were informed that the experiment was part of the campaign iBewust-zijn (Information awareness). With this campaign the Ministry aimed to encourage and support its employees as much as possible in developing knowledge and awareness regarding information security. Also, it reassured the employees that the phishing mail was fake, such that no consequences were attached if subjects indeed had filled in personal details.

## Analysis

Data was collected on whether a subject had clicked on the link and had filled in one or more personal details, and the time of completion. For privacy concerns, the content of what subjects had filled in was not registered. The analysis was conducted on 10,929 observations from the whole sample and on 7,994 observations when division C was excluded.

We measured falling for phishing with three dummy variables: *Visit*, *Fill* and *Fill|Visit*. *Visit* indicates whether the subject clicked on the link and visited the website. Irrespective of whether the subjects filled in personal details, clicking on a link embedded in a phishing email by itself can be very dangerous since such links may infect computers with malware. *Fill* takes value 1 if the subject filled in their password. Although the subjects could also fill in their username or mobile phone number/email address, we chose password as the variable of interest since we regard it as the most confidential data among all and no subjects filled in only the password, supporting the idea that people are more reluctant to give this information away. However, the results are robust to other variables as well since 99.15% of the subjects who filled in any field did so for all three fields asked. Finally, *Fill|Visit* is an indicator variable for whether subjects filled in the password given that they had clicked on the link (hence excluding subjects who did not visit the website). The average of *Fill|Visit* can be interpreted as the probability to fill in the password field conditional on visiting the phishing website. It informs us whether subjects recognized the phishing fraud only after they visited the website.

We performed two types of analysis that were chosen to account for cluster randomized trials (the clusters being basic units described in section 2.2). First, we tested the effectiveness of interventions by weighted t-tests on cluster averages of *Visit*, *Fill* and *Fill|Visit*. For each pair of treatments, the weighted t-test compared the percentages of subjects falling for phishing email of all clusters (basic units) of one treatment with those of the other treatment, but weighting the percentages by the cluster size. Next, we performed logistic regressions on all three variables of interests with standard errors clustered at basic unit level. We also controlled for treatment group, gender, employment contract, organizational division and age.

## Results

### Weighted t-tests

**Fig 1:**
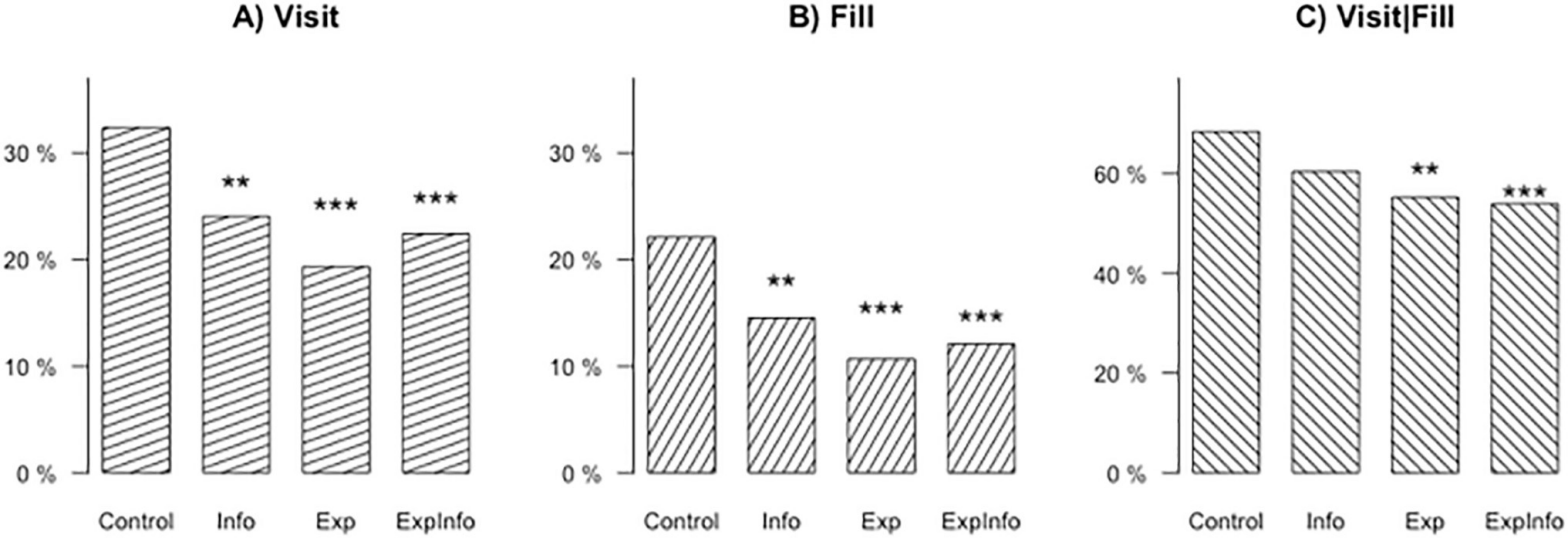
Percentages of subjects falling for phishing email (whole sample). Stars indicating significance levels for difference of each treatment group compared to the control group with * *p* < 0.10, ** *p* < 0.05, *** *p* < 0.01.

**Fig 2:**
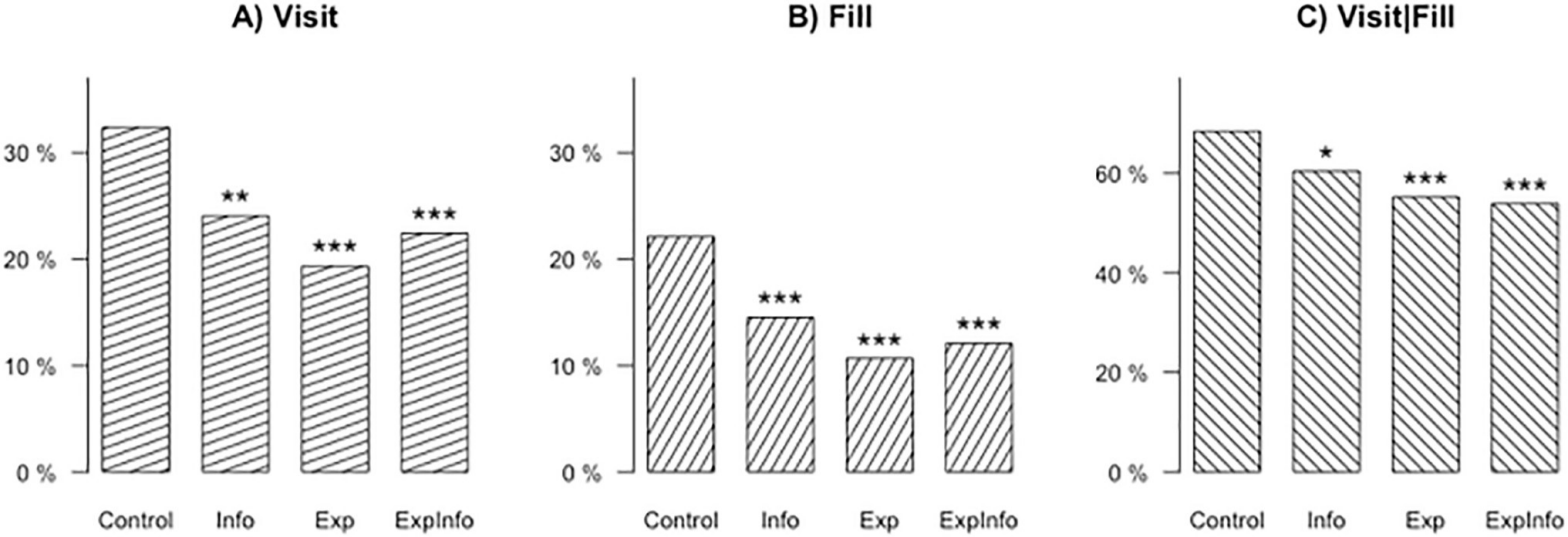
Percentages of subjects falling for phishing email (excluding division C). Stars indicating significance levels for difference of each treatment group compared to the control group with * *p* < 0.10, ** *p* < 0.05, *** *p* < 0.01.

Figs 1 and 2 display, for the whole and reduced sample respectively, the proportion of subjects falling for phishing email in each treatment group for each measure, and reports the significance level of the weighted t-tests. The detailed results of weighted-t tests are given in S3 Table. We describe here the results for the whole sample. Treatment effects are larger when excluding division C. While a third of the subjects in the *Control* group failed to recognize that the received mail was a phishing mail and clicked on the link, the proportion of people visiting the phishing website dropped by 7 to 9 percentage points in the intervention groups.

Among those who visited the link, 68% also filled in their password in the *Control* group. This proportion was very similar in *Info*, being only reduced by 4 points, which was not significant. By contrast, *Fill|Visit* was 58% in *Exp*, and 54% in *ExpInfo*, both significantly lower than in the *Control* group. The difference between *Info* and *ExpInfo* was also significant at a 1% level. The differences between interventions, on the other hand, were not significant with the exception of *Info* and *ExpInfo* in Fig 1C.

The unconditional frequency of subjects filling in their password, as measured by *Fill*, was 22% in *Control*, and dropped to 16%, 14%, and 13% in *Info*, *Exp*, and *ExpInfo* respectively. The effects were stronger after exclusion of Division C. A decrease of 8 percentage points in *Fill* means about 200 fewer passwords given away in each of the intervention treatments than in *Control*. Implementing these simple interventions for an organization of the size of the ministry can avoid that 800 passwords are given to hackers.

### Regression analysis

The results above gave us a first glimpse on the effect of interventions on tackling phishing fraud. As explained above, randomization was only done at the level of basic units, and the size of those varied across organizational divisions. Moreover, as described in section “Group formation” we found some differences between groups in some descriptive statistics when division C was excluded. To account for these differences, we additionally ran logistic regressions with clustered standard errors, controlling for descriptive variables and divisions. This second type of analysis also informed us on the characteristics of the employees who were more prone to click on a dubious link or give away their password. Table 4 presents the average marginal effects of each independent variable on the predicted probability of falling for the phishing email estimated on observation values. We discuss below the effects for the whole sample. The effect sizes were larger after excluding division C and we only describe them when they lead to different conclusions. As a robustness check we ran panel logistic regression with random effects using our basic units as panel variable (identifier). Our results were robust to this specification (see S4 Table).

**Table 4:** Logistic regression analysis—Average marginal effects.

	*dy*/*dx*—Whole sample			*dy*/*dx*—excluding division C		
	Visit	Fill	Fill|Visit	Visit	Fill	Fill|Visit
**Treatment**						
*Control* reference						
*Info*	-0.062* (0.026)	-0.056** (0.021)	-0.050 (0.027)	-0.083*** (0.020)	-0.082*** (0.016)	-0.103*** (0.027)
*Exp*	-0.082*** (0.023)	-0.078*** (0.018)	-0.102*** (0.031)	-0.124*** (0.019)	-0.112*** (0.012)	-0.135*** (0.037)
*ExpInfo*	-0.079*** (0.019)	-0.087*** (0.017)	-0.148*** (0.030)	-0.097*** (0.016)	-0.099*** (0.015)	-0.154*** (0.034)
**Gender**						
*male*	0.042*** (0.010)	0.017 (0.011)	-0.041 (0.024)	0.029* (0.012	0.005 (0.013)	-0.054* (0.027)
**Employee contract**						
*Internal Employee*	0.025 (0.019)	-0.000 (0.012)	-0.064* (0.027)	0.028 (0.018)	0.006 (0.012)	-0.048 (0.028)
**Age group**						
*16-25* reference						
*26-35*	0.035 (0.027)	0.027 (0.019)	0.073 (0.100)	0.029 (0.029)	0.001 (0.025)	-0.095 (0.108)
*36-45*	0.093*** (0.027)	0.067*** (0.018)	0.114 (0.090)	0.072* (0.030)	0.024 (0.023)	-0.097 (0.085)
*46-55*	0.147*** (0.027)	0.118*** (0.017)	0.192* (0.089)	0.131*** (0.031)	0.075** (0.024)	-0.025 (0.083)
*>55*	0.147*** (0.031)	0.138*** (0.022)	0.264** (0.094)	0.122*** (0.033)	0.091** (0.028)	0.063 (0.097)
**Division dummies**	Yes	Yes	Yes	Yes	Yes	Yes
Observations	10929	10929	2869	7994	7994	1947

Division dummies are added with division B as reference category.

Standard errors in parentheses

* *p* < 0.05,

** *p* < 0.01,

*** *p* < 0.001

There is strong evidence that all treatments have a significant negative effect. The probability of visiting the phishing website decreased by around 6 points in the *Info* treatment and 8 points with the other two interventions. The marginal effects of the three interventions were about the same when we studied the probability to fill in the password (see column *Fill*) instead of the probability to click on the link (column *Visit*). The probability to fill in a password conditional on visiting the website was reduced by 10 points in the *Exp* group and by 15 points in the *ExpInfo* group. The effect of *Info* on *Fill|Visit* was not significant on the whole sample, but it was when excluding division C. We tested the differences between the treatment effects and found no significant difference, with three exceptions. The effect of *Info* on *Fill|Visit* was smaller than that of *ExpInfo* in the whole sample and the effects of *Info* on *Fill* and on *Visit* were smaller than those of *Exp* after excluding division C (see S5 Table).

We found some evidence that men were more likely to click on the phishing link but less likely to fill in their password afterwards once they were on the website. Even if men were not more likely than women to give away their password overall (no gender effect on *Fill*), their propensity to click on phishing links could pose a threat when such links trigger malware download. The younger age group (16-25) was the least likely to visit the phishing website. Employees between 36 and 45 were 9% more likely to click on the link than the 16-25 age group and those above 46 were almost 15% more likely to click on the link than the youngest group. The effect of age on the probability to fill in the password conditional on visiting the website were not robust to excluding division C and we therefore refrain from commenting them.

## Discussion

In our field experiment, we observed a non-negligible proportion of subjects falling into a phishing attack. An information campaign substantially reduced that risk, and letting subjects experience a phishing email tended to be at least as effective. Personal experience may have led people to see threats as more probable, and to view themselves as potential future victims, suggesting that cyber-risk beliefs were the most serious barrier to phishing detection. Moreover, people may be more likely to read phishing and/or debriefing emails than information emails. We were not able to investigate this because we could not check whether subjects opened/read the emails we sent. We cannot exclude that the experience intervention was effective because it made employees believe that their employer could know whether they easily give away their password.

It has been argued that information becomes more relevant if it is given after individuals experience the phishing email. Many suggested that combining the two types of intervention yields the best results by reinforcing the effect of each other [14, 15, 19, 20]. Surprisingly, in our experiment, the effectiveness of combining both experience and information did not differ from the effectiveness of experience alone. A possible explanation was that the experience intervention increased employees’ perceived risk enough for them to acquire knowledge on their own or activated previously existing knowledge. Another possible explanation for the absence of synergy is the existence of a ceiling effect. It may be difficult to reduce the proportion of password given away much below 10% with simple interventions as ours. Our results question whether it is worth piling up interventions against phishing fraud. Each intervention requires sending several emails to users. In organizations in which people complain about getting too many emails and cognitive overload, one may prefer to focus on the most effective intervention.

*Info* and *Exp* differed in terms of content (infographics versus a phishing email) but also in terms of dates in which this content was sent. It allowed us to combine both interventions in *Exp* but it decreased the comparability between *Info* and *Exp*. We should therefore be careful when comparing the effects of these two treatments. If anything, we would have expected the *Info* treatment, with more emails and those being sent closer in time to the final phishing email, to be more effective than the *Exp* treatment. This is not what was found. However, it is sound to compare the effect of infographics on its own with the effect of infographics when combined with experience. We expected the latter to be larger, but it was not the case.

The *Exp* treatment involved a first phishing email followed by a debriefing email. We dubbed the corresponding intervention ‘experience’ but it involved both experience and feedback. Our feedback was rather limited (see S4 Fig) and independent of whether people had visited the phishing website and whether they had given their password. Further research could vary the degree of feedback and how personalized it is.

We have interpreted filling in the password field as giving away their password but we cannot know whether they provided their true password or not. What they provided was not saved, for privacy and safety reasons, and we did not have access to their true password anyhow. It could be that some people filled in fake information. We should therefore be cautious with this part of the results. Even if the provided information was not correct, having visited the link already posed a threat. Visiting fraudulent links makes people susceptible to malware attacks and this behavior should be eliminated in the first place.

The treatment effects we observed were slightly lower for the analysis on the whole sample than after excluding division C. In division C, a message was posted online after treatments *Exp* and *ExpInfo* received the first phishing email, thereby affecting the *Control* and *Info* groups as well. If anything, the results on the whole sample give us the lower bound. The effectiveness of experience could also be studied, but in a less controlled way, by comparing the proportion of subjects falling for phishing in the first email and the second email in the two treatments in which two phishing emails were sent. However, this would only be possible if the tests were identical. About 15% of subjects in *Exp* and *ExpInfo* groups clicked on the link in the first email while more than 20% did so in the second email. The difference can come from the second email being more difficult to detect than the first one but also from sending the debriefing email earlier after the first phishing email than after the second. By contrast, comparing the *Exp* and *ExpInfo* treatments with the control as we did in the result section does not suffer from such possible confounds.

Making people more aware of phishing threats and asking them to report suspicious emails may backfire in high number of false positives, i.e., employees misjudging genuine emails and reporting them to the IT department. Kleitman et al. [26] studied which characteristics influence phishing susceptibility but also false positives. We do not have evidence about false positives in our experiment. The operations department of the ministry, in charge of information security, did not report that it was a problem at the time of the study. The official policy was that people should report any doubtful email, the cost of a false positive being judged much lower than that of successful phishing. However, this reasoning was based on the experience of the operations department and their cost-benefit analysis at the time of the experiment. In other instances, anti-phishing campaigns may create a burden on IT departments and generate other organizational costs if the rate of false positives upsurges.

## Conclusion

In a field experiment, we studied the effect of experience, information, and their combination on employees’ reaction to phishing emails. Our information treatment was designed to have a maximal impact, with infographics and clear messages. We could expect the infographics to be especially effective after a first (simulated) phishing experience. Each intervention in isolation had a large effect on the probability to click on a dubious link and to give away personal details. Combining both interventions did not substantially increase the effect of experience alone, even though subjects in the experience treatment were only exposed to one experience. Our results question the opportunity of piling up (costly) interventions.

## Supporting information

S1 Appendix Additional information about the method. (PDF)

S1 Table Kruskal-Wallis test results for equality of groups formed. (PDF)

S2 Table Percentages of subjects falling for phishing email. (PDF)

S3 Table Weighted t-test results. (PDF)

S4 Table XTLogistic regression analysis—Average marginal effects. (PDF)

S5 Table *χ*^2^ test for differences of groups in logistic regression. (PDF)

S1 Fig First phishing email. Translated from Dutch.
(PDF)

S2 Fig First phishing email linked website. Translated from Dutch.
(PDF)

S3 Fig First phishing email Pop-up screen. Translated from Dutch.
(PDF)

S4 Fig Short debriefing after first phishing mail. Translated from Dutch.
(PDF)

S5 Fig Infographics. Translated from Dutch and contact details are replaced for privacy concerns.
(PDF)

S6 Fig Second phishing email. Translated from Dutch.
(PDF)

S7 Fig Second phishing email linked website. Translated from Dutch.
(PDF)

S8 Fig Second phishing email Pop-up screen. Translated from Dutch.
(PDF)

## References

[1] Vishwanath A, Herath T, Chen R, Wang J, Rao HR. Why do people get phished? Testing individual differences in phishing vulnerability within an integrated, information processing model. Decision Support Systems. 2011; 51(3): 576–586. 10.1016/j.dss.2011.03.002

[2] Ramanathan V, Wechsler H. Phishing detection and impersonated entity discovery using Conditional Random Field and Latent Dirichlet Allocation. Computers & Security. 2013; 34: 123–139. 10.1016/j.cose.2012.12.002

[3] Blythe M, Petrie H, Clark JA. F for fake: Four studies on how we fall for phish. In: Proceedings of the SIGCHI Conference on Human Factors in Computing Systems. ACM; 2011. p. 3469–3478.

[4] Berghel H. Phishing mongers and posers. Communications of the ACM. 2006; 49(4): 21–25.

[5] Gupta Brij B and Arachchilage Nalin AG and Psannis Kostas E. Defending against phishing attacks: taxonomy of methods, current issues and future directions. Telecommunication Systems. 2018; 67(2): 247–267. 10.1007/s11235-017-0334-z

[6] Ransbotham S, Mitra S. Choice and chance: A conceptual model of paths to information security compromise. Information Systems Research. 2009; 20(1): 121–139. 10.1287/isre.1080.0174

[7] Bada M, Sasse A, Nurse JRC. Cyber Security Awareness Campaigns: Why do they fail to change behaviour? In: International Conference on Cyber Security for Sustainable Society; 2015. p. 118–131.

[8] Pahnila S, Siponen M, Mahmood A. Employees’ behavior towards IS security policy compliance. In: System sciences, 2007. HICSS 2007. 40th Annual Hawaii International Conference on. IEEE; 2007. p. 156b–156b.

[9] Vroom C, von Solms R. Towards information security behavioural compliance. Computers & Security. 2004; 23(3): 191–198. 10.1016/j.cose.2004.01.012

[10] Bulgurcu B, Cavusoglu H, Benbasat I. Information security policy compliance: An empirical study of rationality-based beliefs and information security awareness. MIS quarterly. 2010; 34(3): 523–548. 10.2307/25750690

[11] Whitman M, Mattord H. Principles of Information Security: Thompson Course Technology. Kennesaw State University 2003.

[12] Albrechtsen E. A qualitative study of users’ view on information security. Computers & security. 2007; 26(4): 276–289. 10.1016/j.cose.2006.11.004

[13] Cai J, Song C. Do disaster experience and knowledge affect insurance take-up decisions? Journal of Development Economics. 2017; 124: 83–94. 10.1016/j.jdeveco.2016.08.007

[14] Kumaraguru P, Rhee Y, Acquisti A, Cranor LF, Hong J, Nunge E. Protecting people from phishing: The design and evaluation of an embedded training email system. In: Proceedings of the SIGCHI conference on Human factors in computing systems. ACM; 2007. p. 905–914.

[15] Sheng S, Magnien B, Kumaraguru P, Acquisti A, Cranor LF, Hong J, et al. Anti-phishing phil: The design and evaluation of a game that teaches people not to fall for phish. In: Proceedings of the 3rd symposium on Usable privacy and security. ACM; 2007. p. 88–99.

[16] Sheng S, Holbrook M, Kumaraguru P, Cranor LF, Downs J. Who falls for phish?: A demographic analysis of phishing susceptibility and effectiveness of interventions. In: Proceedings of the SIGCHI Conference on Human Factors in Computing Systems. ACM; 2010. p. 373–382.

[17] Downs JS, Holbrook MB, Cranor LF. Decision strategies and susceptibility to phishing. In: Proceedings of the second symposium on Usable privacy and security. ACM; 2006. p. 79–90.

[18] Wright RT, Marett K. The influence of experiential and dispositional factors in phishing: An empirical investigation of the deceived. Journal of Management Information Systems. 2010; 27(1): 273–303. 10.2753/MIS0742-1222270111

[19] Bowen BM, Devarajan R, Stolfo S. Measuring the human factor of cyber security. In: Technologies for Homeland Security (HST), 2011 IEEE International Conference on. IEEE; 2011. p. 230–235.

[20] Burns MB, Durcikova A, Jenkins JL. What kind of interventions can help users from falling for phishing attempts: A research proposal for examining stage-appropriate interventions. In: System Sciences (HICSS), 2013 46th Hawaii International Conference on. IEEE; 2013. p. 4023–4032.

[21] Mohebzada J, El Zarka A, BHojani AH, Darwish A. Phishing in a university community: Two large scale phishing experiments. In: Innovations in Information Technology (IIT), 2012 International Conference on. IEEE; 2012. p. 249–254.

[22] Williams Emma J., Hinds Joanne, and Joinson Adam N. Exploring susceptibility to phishing in the workplace. International Journal of Human-Computer Studies. 2018; 120: 1–13. 10.1016/j.ijhcs.2018.06.004

[23] Silic M, Back A. The dark side of social networking sites: Understanding phishing risks. Computers in Human Behavior. 2016;60:35–43. 10.1016/j.chb.2016.02.050

[24] Halevi T, Memon N, Nov O. Spear-phishing in the wild: A real-world study of personality, phishing self-efficacy and vulnerability to spear-phishing attacks. 2015; 10.2139/ssrn.2544742.

[25] Siadati H, Palka S, Siegel A, McCoy D. Measuring the Effectiveness of Embedded Phishing Exercises. In: 10th USENIX Workshop on Cyber Security Experimentation and Test (CSET 17). USENIX Association; 2017.

[26] Kleitman Sabina and Law Marvin KH and Kay Judy. It’s the deceiver and the receiver: Individual differences in phishing susceptibility and false positives with item profiling. PloS one. 2018; 13(10): e0205089 10.1371/journal.pone.0205089 30365492 PMC6203253

[27] Vishwanath Arun, Harrison Brynne, and Yu Jie Ng. Suspicion, cognition, and automaticity model of phishing susceptibility. Communication Research. 2018; 45(8): 1146–1166. 10.1177/0093650215627483

[28] Musuva Paula MW and Getao Katherine W and Chepken Christopher K. A new approach to modelling the effects of cognitive processing and threat detection on phishing susceptibility. Computers in Human Behavior. 2019; 94: 154–175. 10.1016/j.chb.2018.12.036

[29] Zigmont Jason J., and Kappus Liana J., and Sudikoff Stephanie N. Theoretical foundations of learning through simulation. Seminars in perinatology. 2011; 35(2): 47–51. 10.1053/j.semperi.2011.01.002 21440810

[30] Xiong Aiping and Proctor Robert W and Yang Weining and Li Ninghui. Embedding Training Within Warnings Improves Skills of Identifying Phishing Webpages. Human factors. 2019; 61(4): 577–595. 10.1177/0018720818810942 30526089

[31] Arachchilage N. A. G., and Love S. Security awareness of computer users: A phishing threat avoidance perspective. Computers in Human Behavior. 2014; 38: 304–312. 10.1016/j.chb.2014.05.046

[32] Alsharnouby M., Alaca F., and Chiasson S. Why phishing still works: User strategies for combating phishing attacks. International Journal of Human-Computer Studies. 2015; 82: 69–82. 10.1016/j.ijhcs.2015.05.005

