# Supplementary material for: Informing, simulating experience, or both: A field experiment on phishing risks
Source: PLoS One. 2019 Dec 18;14(12):e0224216. doi: 10.1371/journal.pone.0224216 (PMC6919577; doi:10.1371/journal.pone.0224216)
Supplement: S1 Appendix — (PDF) [file pone.0224216.s001.pdf]

Additional information about the method

Subjects

The total number of employees in the Dutch Ministry of Economic Affairs was 12,567. We excluded those employees with errors/flaws in the dataset, i.e. 40 employees due to their age (age < 16 & age > 70), 17 due to missing organizational unit information and 408 due to missing e-mail addresses. Furthermore, we excluded the departments which were covered by a different IT assistance. Finally, some employees were excluded due to their rank in the organization (Minister, State Secretary and Secretary General etc.).

Pre-intervention

We made arrangements with several parties such as the IT helpdesk and the information security coordinators in case subjects contacted them during the experiment. We wanted to assure that none of these parties would inform the subjects that they were part of an experiment, because this could confound our results. Therefore, we provided them with standardized protocols for email and phone. This allowed these parties to answer the possible questions of subjects, without disturbance of the experiment.

Moreover, at the IT helpdesk a dedicated group of employees was formed, who were in first line of contact with the subjects. Subjects were redirected to this dedicated group through a choice option at the service line or by automatically forwarding emails to them. When subjects phoned or emailed them, they were told standardized answers, which were carefully constructed and suited for each specific question participants could ask. This way, we could; (1) inform/help subjects as much as possible, without informing them of the experiment, (2) control the outgoing messages.

T = 1

Participants who recognized the mail as being fraudulent and send it to the IT helpdesk

(of the ministry of Economic Affairs), received an answer stating that the mail was indeed a phishing email, that the threat had receded, and that no further actions on behalf of the user were needed (Appendix **??**). If participants mailed the IT helpdesk, without adding the mail, they were still asked to send the phishing email. This was to establish with certainty that the notification indeed concerned our 'imitation' phishing email and not a 'real-non-experiment-related' phishing email. Employees who phoned the IT helpdesk to report the 'imitation' phishing email, were first asked the subject and sender of the email. Furthermore, if it indeed concerned our email, they were asked to send the email as an attachment in an email to the IT helpdesk. Employees who did not see the email as suspicious but asked substantive questions regarding the 'mobile password recovery system' were given the answer that they would receive an answer within three workdays (which is standard protocol for all IT related questions).

T = 2, 3, 4

The first information email starts with a short introduction of the director of operational management. The introduction states that the received mail is part of an information provision campaign, consisting of three information emails. Furthermore the subjects are given information about how phishing fraud works and the newer generation of phishing fraud, spear phishing attacks.

The second information mail starts with a short recap of the first information mail. Furthermore it provides the reader with six points of recognition by which he/she could determine whether emails are fraudulent or not, such as; (1) the mail address of the sender, (2) the salutation, (3) style of writing (grammatical or spelling errors), (4) the hyperlink in the mail, (5) look the sender up on the internet, (6) check the mail address in the signature. Also it lists the types of information that the ministry never will ask their employees. Finally, it states the three largest consequences (for organizations) of phishing fraud.

The third information mail starts again with a short recap of the first two information mails. Furthermore it provides information about, how you should act in the case that (you

think that) you have received a phishing mail and how and where you could report a phishing mail. It concludes with some useful links to other (phishing awareness) campaigns of the Dutch Ministry of Economic Affairs (www.veiliginternetten.nl and iBewustzijn) where more information could be found, including some examples of phishing mails.

Moreover the three infographics had some synergetic power, as each of the three infographics starts with a short recap of the previous mail(s). This ensures the participants that each individual mail is understandable in itself, without necessity of reading them all. Furthermore, the infographics; (1) were readable on the mobile phone, laptop and tablet, (2) did not need to be downloaded first, and (3) were in line with the visual identity style of the Ministry. The story based graphics and annotations, were highly related to the core business activities of the employees and all images/pictures were copyright free, bought or self-made.
