# Supplementary material for: Informing, simulating experience, or both: A field experiment on phishing risks
Source: PLoS One. 2019 Dec 18;14(12):e0224216. doi: 10.1371/journal.pone.0224216 (PMC6919577; doi:10.1371/journal.pone.0224216)
Supplement: S1 Table — (PDF) [file pone.0224216.s002.pdf]

	Whole sample		Excluding div. C	
Attribute	χ^2	p	χ^2	p
Age	2.479	0.479	28.645	0.000
Age-Group	3.697	0.296	26.179	0.000
Gender	0.508	0.917	5.544	0.136
Employee Contract	0.932	0.818	6.822	0.078
Organisational division	21.256	0.000	15.274	0.002
