# Supplementary material for: Informing, simulating experience, or both: A field experiment on phishing risks
Source: PLoS One. 2019 Dec 18;14(12):e0224216. doi: 10.1371/journal.pone.0224216 (PMC6919577; doi:10.1371/journal.pone.0224216)
Supplement: S2 Table — (PDF) [file pone.0224216.s003.pdf]

	Whole Sample			Excluding div. C		
	Visit	*Fill*	*Fill|Visit*	*Visit*	*Fill*	*Fill|Visit*
Control	32.21%	21.78%	67.62%	32.39%	22.13%	68.32%
Info	25.58%	16.28%	63.62%	24.06%	14.53%	60.38%
Exp	23.31%	13.58%	58.27%	19.34%	10.67%	55.21%
ExpInfo	23.92%	12.80%	53.51%	22.43%	12.09%	53.90%
