# Supplementary material for: Informing, simulating experience, or both: A field experiment on phishing risks
Source: PLoS One. 2019 Dec 18;14(12):e0224216. doi: 10.1371/journal.pone.0224216 (PMC6919577; doi:10.1371/journal.pone.0224216)
Supplement: S3 Table — (PDF) [file pone.0224216.s004.pdf]

(a) Whole sample

Visit

	Info		*Exp*		*ExpInfo*	
	t	p	t	p	t	p
Control	2.220	0.032	3.723	0.000	3.830	0.000
Info			0.747	0.459	0.579	0.566
Exp					-0.273	0.786

Fill

	Info		*Exp*		*ExpInfo*	
	t	p	t	p	t	p
Control	2.361	0.023	4.328	0.000	5.116	0.000
Info			1.163	0.251	1.577	0.123
Exp					0.450	0.654

Fill|Visit

	Info		*Exp*		*ExpInfo*	
	t	p	t	p	t	p
Control	1.103	0.276	2.542	0.014	4.176	0.000
Info			1.253	0.216	2.707	0.009
Exp					1.486	0.142

(b) Excluding div. C

Visit

	Info		*Exp*		*ExpInfo*	
	t	p	t	p	t	p
Control	2.642	0.012	5.159	0.000	3.971	0.000
Info			1.619	0.115	0.563	0.577
Exp					-1.404	0.167

Fill

	Info		*Exp*		*ExpInfo*	
	t	p	t	p	t	p
Control	3.227	0.003	5.781	0.000	4.794	0.000
Info			1.909	0.065	1.145	0.259
Exp					-0.829	0.411

Fill|Visit

	Info		*Exp*		*ExpInfo*	
	t	p	t	p	t	p
Control	1.870	0.070	2.767	0.009	3.618	0.001
Info			0.942	0.352	1.604	0.116
Exp					0.581	0.564
