# Supplementary material for: Informing, simulating experience, or both: A field experiment on phishing risks
Source: PLoS One. 2019 Dec 18;14(12):e0224216. doi: 10.1371/journal.pone.0224216 (PMC6919577; doi:10.1371/journal.pone.0224216)
Supplement: S4 Table — (PDF) [file pone.0224216.s005.pdf]

	dy/dx - All observations			dy/dx - excluding NVMA		
	Visit	Fill	Fill|Visit	Visit	Fill	Fill|Visit
**Treatment**						
Control omitted						
Info	-0.079^{*}	-0.069^{*}	-0.056	-0.096^{*}	-0.096^{**}	-0.120^{**}
	(0.037)	(0.029)	(0.041)	(0.043)	(0.033)	(0.043)
Exp	-0.088^{**}	-0.068^{**}	-0.082^{*}	-0.137^{***}	-0.113^{***}	-0.125^{**}
	(0.033)	(0.026)	(0.038)	(0.040)	(0.031)	(0.041)
ExpInfo	-0.087^{**}	-0.085^{***}	-0.139^{***}	-0.110^{**}	-0.106^{***}	-0.150^{***}
	(0.031)	(0.025)	(0.036)	(0.039)	(0.030)	(0.038)
**Gender**						
male	0.040^{***}	0.015^{*}	-0.040^{*}	0.032^{**}	0.010	-0.049^{*}
	(0.009)	(0.008)	(0.020)	(0.010)	(0.009)	(0.024)
**Employee contract**						
Internal Employee	0.005	-0.014	-0.065^{*}	0.009	-0.006	-0.046
	(0.014)	(0.011)	(0.030)	(0.013)	(0.011)	(0.030)
**Age group**						
16-25 omitted						
26-35	0.022	0.022	0.091	0.031	0.005	-0.089
	(0.024)	(0.016)	(0.085)	(0.030)	(0.024)	(0.110)
36-45	0.082^{***}	0.062^{***}	0.135	0.077^{**}	0.030	-0.089
	(0.024)	(0.016)	(0.081)	(0.030)	(0.023)	(0.107)
46-55	0.133^{***}	0.110^{***}	0.215^{**}	0.134^{***}	0.080^{***}	-0.017
	(0.024)	(0.017)	(0.081)	(0.030)	(0.024)	(0.106)
>55	0.137^{***}	0.132^{***}	0.285^{***}	0.129^{***}	0.098^{***}	0.070
	(0.025)	(0.018)	(0.081)	(0.031)	(0.025)	(0.107)
**Division fixed effects**	Yes	Yes	Yes	Yes	Yes	Yes
Observations	10929	10929	2869	7994	7994	1947

Division fixed effects are added while base division is B.

Standard errors in parentheses

* $p < 0.05$, ** $p < 0.01$, *** $p < 0.001$
