# Supplementary material for: Informing, simulating experience, or both: A field experiment on phishing risks
Source: PLoS One. 2019 Dec 18;14(12):e0224216. doi: 10.1371/journal.pone.0224216 (PMC6919577; doi:10.1371/journal.pone.0224216)
Supplement: S5 Table — (PDF) [file pone.0224216.s006.pdf]

(a) Whole sample

Visit

	Exp		*ExpInfo*	
	χ^2	p	χ^2	p
Info	0.51	0.475	0.47	0.493
Exp			0.02	0.888

Fill

	Exp		*ExpInfo*	
	χ^2	p	χ^2	p
Info	1.08	0.299	2.49	0.115
Exp			0.33	0.565

Fill|Visit

	Exp		*ExpInfo*	
	χ^2	p	χ^2	p
Info	1.92	0.166	7.41	0.007
Exp			1.43	0.232

(b) Excluding div. C

Visit

	Exp		*ExpInfo*	
	χ^2	p	χ^2	p
Info	3.68	0.055	0.54	0.461
Exp			2.07	0.150

Fill

	Exp		*ExpInfo*	
	χ^2	p	χ^2	p
Info	4.87	0.027	1.26	0.262
Exp			0.86	0.354

Fill|Visit

	Exp		*ExpInfo*	
	χ^2	p	χ^2	p
Info	0.52	0.472	1.58	0.209
Exp			0.16	0.693
