# Supplementary material for: Informing, simulating experience, or both: A field experiment on phishing risks
Source: PLoS One. 2019 Dec 18;14(12):e0224216. doi: 10.1371/journal.pone.0224216 (PMC6919577; doi:10.1371/journal.pone.0224216)
Supplement: S1 Fig — Translated from Dutch. (PDF) [file pone.0224216.s007.pdf]

Sender: BusinessOperations@Mlnez.nl
Subject: Activate your personal Mobile Password Recovery System

Dear Economic Affairs colleague,

After a successful pilot of the department Business Operations, we recently started implementing the EZ - Mobile Password Recovery System (EZ-MPRS) for all EA employees. Via this system you can, at all times, retrieve and change your password for your user account. With this, we hope to serve you even better and faster.

Our data shows that you are not yet using the EZ - Mobile Password Recovery System. That is why we ask you, to link your account to your mobile number.

Activate here your EZ – Mobile Password Recovery System (MPRS)

For more information, see link below:

https://rijksweb.nl/ezmprs

Best regards,

Director Business Operations

Dit bericht kan informatie bevatten die niet voor u is bestemd. Indien u niet de geadresseerde bent of dit bericht abusievelijk aan u is gezonden, wordt u verzocht dat aan de afzender te melden en het bericht te verwijderen.
De Staat aanvaardt geen aansprakelijkheid voor schade, van welke aard ook, die verband houdt met risico's verbonden aan het elektronisch verzenden van berichten.

This message may contain information that is not intended for you. If you are not the addressee or if this message was sent to you by mistake, you are requested to inform the sender and delete the message.
The State accepts no liability for damage of any kind resulting from the risks inherent in the electronic transmission of messages.
