# Supplementary material for: Informing, simulating experience, or both: A field experiment on phishing risks
Source: PLoS One. 2019 Dec 18;14(12):e0224216. doi: 10.1371/journal.pone.0224216 (PMC6919577; doi:10.1371/journal.pone.0224216)
Supplement: S2 Fig — Translated from Dutch. (PDF) [file pone.0224216.s008.pdf]

EZ - MPRS
Ministerie van Economische Zaken

EZ - Mobile Password Recovery System (MPRS) 1/2

Link in two simple steps your useraccount to your mobile phone number

Username *	
Password *	
Mobile number *	
	Send

* Indicates required field
