# Supplementary material for: Informing, simulating experience, or both: A field experiment on phishing risks
Source: PLoS One. 2019 Dec 18;14(12):e0224216. doi: 10.1371/journal.pone.0224216 (PMC6919577; doi:10.1371/journal.pone.0224216)
Supplement: S3 Fig — Translated from Dutch. (PDF) [file pone.0224216.s009.pdf]

EZ - MPRS
Ministerie van Economische Zaken

EZ - Mobile Password Recovery System (MPRS) 2/2

Thanks for your registration. Your request is being processed. You will receive confirmation of your registration by email within 5 working days.

Business Operations / DICTU ServiceDesk
