# Supplementary material for: Informing, simulating experience, or both: A field experiment on phishing risks
Source: PLoS One. 2019 Dec 18;14(12):e0224216. doi: 10.1371/journal.pone.0224216 (PMC6919577; doi:10.1371/journal.pone.0224216)
Supplement: S4 Fig — Translated from Dutch. (PDF) [file pone.0224216.s010.pdf]

Ministerie van Economische Zaken

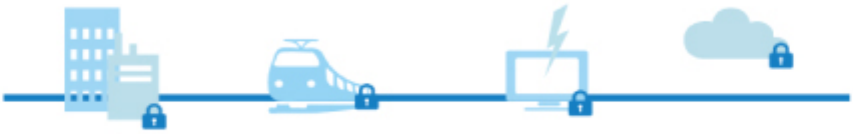

'Imitation' Phishing email

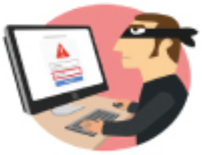

You received today an 'Imitation' phishing email, with sender Business Operations. This email, with subject EZ Mobile Password Recovery System, was an 'Imitation' phishing email, designed to increase your awareness on the topic of phishing. This way we can all contribute to a safer digital working environmnet. Via phishing emails, malicious people can abuse your (personal) data.

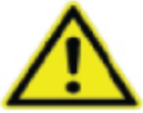

If you have filled in your password, change this immediately! *This can be done by using the key combination; Control + ALT + DEL, and then choose; "Change Password". Despite taken security measures, safety risks can never be entirely ruled out. If you have forwarded the phishing email to someone, we ask you to inform that person (excluding DICTU Servicedesk and xxx@xxx.nl).*

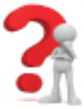

We thank you for your contribution to a safer digital work environment. To approach the situation of a phishing email as realistically as possible, it was decided not to inform you in advance. We ask for your understanding. For questions about this 'imitation' phishing email, please contact the *IB-Coordinator* of your department.
