# Supplementary material for: Informing, simulating experience, or both: A field experiment on phishing risks
Source: PLoS One. 2019 Dec 18;14(12):e0224216. doi: 10.1371/journal.pone.0224216 (PMC6919577; doi:10.1371/journal.pone.0224216)
Supplement: S5 Fig — Translated from Dutch and contact details are replaced for privacy concerns. (PDF) [file pone.0224216.s011.pdf]

Ministerie van Economische Zaken

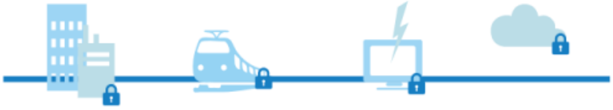

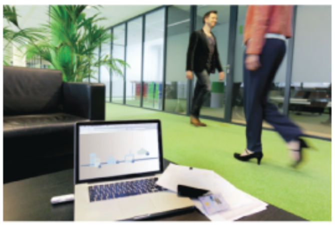

Dear Colleagues,

Last year october, the department Business Operations organized the kickoff of the campaign iAwareness at the Ministry of Economic Affairs (EA); a huge success!

Aim of the campaign was to train your digital skills and to improve your knowlegde on prevention and recognition of security risks. In line with this campaign, this month, you will receive three informative mails on the topic of 'phishing'.

Please read these emails with care to improve your digital skills on the topic of: (1) What is phishing?, (2) How to to recognize phishing emails?, and (3) What to do when you think/know you received a phishing email?

More information of iAwareness can be found on:
https://www.ibewustzijnoverheid.nl

Best regards,

Managing Director EA

Digital Skills - Phishing emails (1)

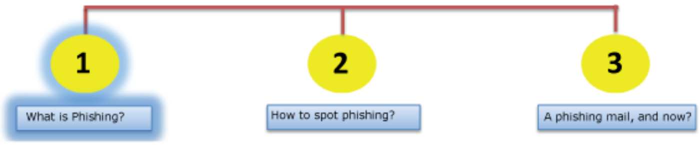

What is phishing?

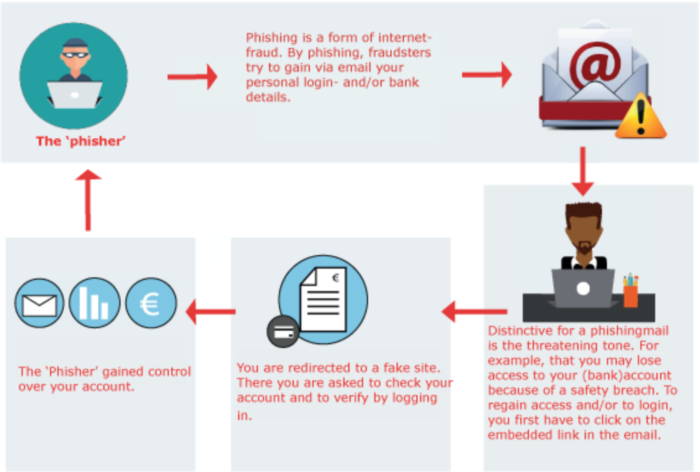

Phishing via Social Media

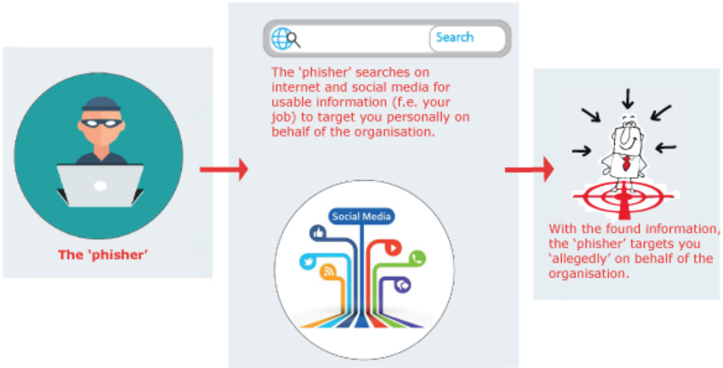

Ministerie van Economische Zaken

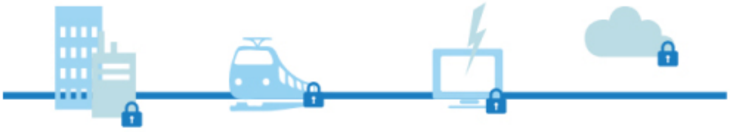

Digital Skills - Phishing emails (2)

1 — Wat is phishing?

2 — How to spot phishing?

3 — A phishing mail, and now?

Phishing is a form of internet-fraud. By phishing, fraudsters try to gain via email your personal login- and/or bank details.

How to spot phishing?

Van: BusinessOperations@Minez.nl (1)
Aan: Employee@minez.nl
CC:
Onderwerp: Activate your personal EA-Mobile Password Recovery System

Dear Economic Affairs colleague , (2)

After a successful pilot of the department Business Operations, we recently started implementing the EZ - Mobile Password Recovery System (EZ-MPRS) for all EA employees. Via this system you can, at all times, retrieve and change your password for your user account. With this, we hope to serve you even better and faster. (3)

Our data shows that you are not yet using the EZ - Mobile Password Recovery System. That is why we ask you, to link your account to your mobile number.

Activate here your EZ – Mobile Password Recovery System (MPRS) (4)

For more information, see link below:

http://rijksweb.nl/ezmprs (4)

Best regards,
Director Business Operations

1. Check (typos in) the email address
   - A reliable sender does not uses gmail/hotmail, to verify account details. Be sure to look at the email address to confirm the true sender.
2. Check the salutation
   - Phishing emails often have an impersonal salutation.
3. Check the writing style
   - Attackers are often less concerned about spelling or being grammatically correct than a normal normal sender would be.
4. Check the link
   - Hover or mouse over the link without clicking anything. If the alt text looks strange or doesn't match what the link descritption says, don't click on it.
   - Sites where you have to log in, generally do not send links asking you to log in. If you have to log in, just type the address of the website in the address bar yourself.

Check the email signature
- Is it the true email address of the sender?

Check the sender
- Do you know the sender? Google his or her name to check if the sender really exists.

! Attention: Your employer will never aks your personal - and/or login details via email.
Neither ask you via a link in an email to check and verify accountdetails.
If you receive such an email, please take appropriate measures.

Consequences of phishingmails

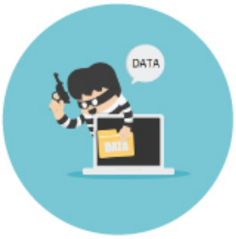

Loss of confidential and state secret information.

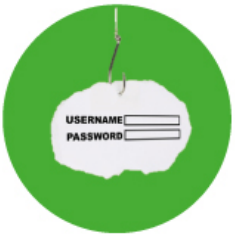

Loss of personal detail such as name, email adress and (login) accountdetails of the EA- netwerk, het Governmental portal DoMuS

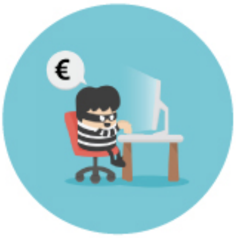

Financial loss

Ministerie van Economische Zaken

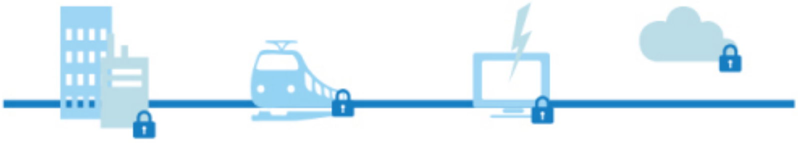

Digital Skills - Phishing emails (3)

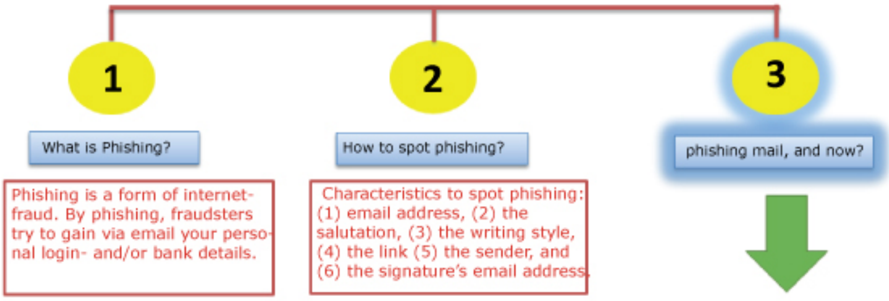

How to respond to a (potential) phishing email?

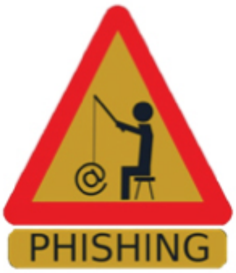

- Do NOT click on the embedded link.
- Never share login details or confidential Information.
- Send the email as attachment to **xxxx@xxx.nl**.
- **RVO:** CC to: xxxx2@xxx.nl
- **NVWA:** CC to xxxx3@xxx.nl

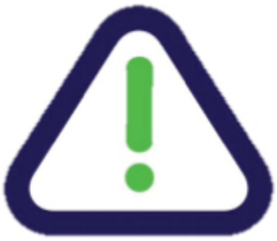

Questions regarding suspicious mails? - Contact details:

- **DICTU Servicedesk**

**Phone number:**	xxx xxx 6666<br>xxx xxx 8888	(66666)<br>(8888)	**voor:**<br>**voor:**	KD & SODM<br>RVO & NVWA<br>AT & DICTU
**Email address:**	**xxxx4@xxx.nl**			

- **The IB - Coordinator(s) of your department**
- **Portal (Informationsecurity)**
  - Intranet of your department

More Information?

- https://www.ibewustzijnoverheid.nl
- https://veiliginternetten.nl
