# Supplementary material for: Informing, simulating experience, or both: A field experiment on phishing risks
Source: PLoS One. 2019 Dec 18;14(12):e0224216. doi: 10.1371/journal.pone.0224216 (PMC6919577; doi:10.1371/journal.pone.0224216)
Supplement: S6 Fig — Translated from Dutch. (PDF) [file pone.0224216.s012.pdf]

Sender: helpdesk@dlctu.nl
Subject: Increase your maximum outlook exchange storage limit.

Dear Economic Affairs colleague,

Your mailbox has exceeded the maximum storage limit set by DICTU. You may not send or receive e-mail until you have upgraded the maximum limit. To increase your limit, click on the link below:

Increase your storage limit here

If you do not do this, you run the risk that your mail account will be locked. Thank you for your cooperation.

Best regards,

For more information, see the link below:

https://rijksweb.nl/exchangelimiet

The DICTU Helpdesk

Dit bericht kan informatie bevatten die niet voor u is bestemd. Indien u niet de geadresseerde bent of dit bericht abusievelijk aan u is gezonden, wordt u verzocht dat aan de afzender te melden en het bericht te verwijderen. De Staat aanvaardt geen aansprakelijkheid voor schade, van welke aard ook, die verband houdt met risicos verbonden aan het elektronisch verzenden van berichten.

This message may contain information that is not intended for you. If you are not the addressee or if this message was sent to you by mistake, you are requested to inform the sender and delete the message. The State accepts no liability for damage of any kind resulting from the risks inherent in the electronic transmission of messages.
