# Supplementary material for: Informing, simulating experience, or both: A field experiment on phishing risks
Source: PLoS One. 2019 Dec 18;14(12):e0224216. doi: 10.1371/journal.pone.0224216 (PMC6919577; doi:10.1371/journal.pone.0224216)
Supplement: S7 Fig — Translated from Dutch. (PDF) [file pone.0224216.s013.pdf]

Microsoft®
Exchange

Microsoft Exchange Outlook

Update the Microsoft Exchange account, to increase your storage limit.

Username *	
Email Address *	
Password *	
	Send

* Indicates required field
