# Supplementary material for: Informing, simulating experience, or both: A field experiment on phishing risks
Source: PLoS One. 2019 Dec 18;14(12):e0224216. doi: 10.1371/journal.pone.0224216 (PMC6919577; doi:10.1371/journal.pone.0224216)
Supplement: S8 Fig — Translated from Dutch. (PDF) [file pone.0224216.s014.pdf]

Microsoft Exchange Outlook

Thank you for updating your account. Your request is being processed. After completion, you will receive confirmation within 5 working days.
